# Effects of hydrolyzed fish protein and autolyzed yeast as substitutes of fishmeal in the gilthead sea bream (*Sparus aurata*) diet, on fish intestinal microbiome

**DOI:** 10.1186/s12917-020-02335-1

**Published:** 2020-04-22

**Authors:** S. Rimoldi, E. Gini, J. F. A. Koch, F. Iannini, F. Brambilla, G. Terova

**Affiliations:** 1 Department of Biotechnology and Life Sciences, University of Insubria, Via J.H. Dunant, 3, 21100 Varese, Italy; 2 Biorigin Brazil. Rua XV de Novembro, 865, Lençóis Paulista, São Paulo 18680-900 Brazil; 3 VRM srl Naturalleva, Via Sommacampagna, 63/D, 37137 Verona, Italy

**Keywords:** Aquaculture, Gut microbiota, Fish protein hydrolysate, autolyzed yeast, Single cell proteins, fish nutrition

## Abstract

**Background:**

This study evaluated the effects of partial substitution of dietary fishmeal (FM) with either fish protein hydrolysate (FPH) or autolysed dried yeast (HiCell®, Biorigin, Brazil) on intestinal microbiota of gilthead sea bream (*Sparus aurata*). A total number of 720 fish of 122.18 ± 6.22 g were fed for 92 days with three different diets in triplicate (3 tanks/diet). A diet based on FM/vegetable meal was used as control. The other two diets were formulated by replacing FM with 5% of either FPH or HiCell®. To analyze the gut microbiota associated to autochthonous and allochthonous microbial communities, the Illumina MiSeq platform for sequencing of 16S rRNA gene and QIIME pipeline were used.

**Results:**

A total number of 102 OTUs (operational taxonomic units) at 97% identity were identified in fish gut samples collected at the end of feeding trial. Fourteen OTUs constituted the core gut microbiota, i.e. those OTUs found in at least nine out of fifteen samples per group and shared regardless of the diet. Eight OTUs were assigned to *Firmicutes* represented by *Lactobacillus*, *Staphylococcus*, and *Bacillus* genera, and six to *Proteobacteria* phylum. Dietary dried yeast autolysate modulated the intestinal microbiota by promoting the growth of some beneficial bacteria. At order level, fish fed yeast showed an enrichment in *Bacillales* and *Clostridiales* as compared to the control group, whereas fish fed FPH showed a significantly lower amount of bacteria belonging to *Alteromonadales* and *Enterobacteriales* than the other two feeding groups. Although we did not observe any effect of 5% FM replacement with alternative nitrogen sources at phylum level, at lower taxonomical levels, the composition of gut microbiota, in terms of relative abundance of specific taxa, was significantly influenced by the dietary treatment.

**Conclusions:**

The metabarcoding analysis revealed a clearly intestinal microbiota modulation in response to dietary autolyzed yeast. The abundance of some beneficial bacteria, i.e. indigestible carbohydrate degrading- and SCFA producing bacteria, was positively affected. Brewer’s yeast autolysate could be a valid alternative protein source to FM as well as a valid functional ingredient for aquafeed production.

## Background

The rapid growth of the aquaculture industry together with finite supplies of fishmeal (FM) makes it necessary to find sustainable alternative protein sources for the aquafeed sector.

Actually, various sources of protein have been considered and tested as alternative ingredients for FM. Among them, plant feedstuffs, mainly soybean meal, soybean concentrate, and grains glutens, are the most commonly used [1–3]. However, due to their amino acid imbalances, presence of anti-nutritional factors, and low palatability, a high FM replacement with vegetable meals is generally not well accepted, especially for carnivorous fish species.

In this regard, single cell proteins (SCP), including microalgae, bacteria, and yeast, represent alternative non-conventional nitrogen sources that are frequently used as feed ingredients for fish being rich in valuable and bioactive components. In particular, yeast is an environmentally friendly and sustainable ingredient due to its ability to convert low-value forest and agricultural biomass residues into high-value feed ingredients and its limited dependence on arable land, water, and climatic conditions. Yeast contains a wide range of bioactive components with potential as functional ingredients, such as α-glucan, β-glucan, α-mannan, nucleic acids, and antioxidants [4]. Brewer’s yeast, mainly *Saccharomyces cerevisiae* strain, have been used as nitrogen-rich ingredient in aquaculture feeds from the beginning of the 1990s [5]. Nutritional yeasts are usually pure yeasts grown under controlled production conditions, cultivated specifically for use as a nutritional supplement and are not by-product of the brewing process [6]. However, in the last years, the increasing sensitivity for sustainable lifestyle based on circular economy is encouraging the development of new technologies to produce nutritional yeasts from low-value and non-food lignocellulosic biomass for use in aquaculture feeds [7].

To nowadays, there are several evidences of a positive effect of yeast or its cell wall components, such as mannan oligosaccharides, glucans and chitin, on fish immune system by stimulating non-specific (innate) cellular and humoral immunity [8, 9]. However, these effects can vary depending on yeast strain, processing technology, and dietary inclusion level [7, 10, 11].

Previous studies established that protein from *S. cerevisiae* yeast can successfully replace up to 50% of fishmeal protein without negative effects on fish growth performance, whereas a dietary inclusion of up to 30% of brewer’s yeast improved feed efficiency [12–14]. However, poor nitrogen digestibility, probably due to the external mannoprotein cell wall, can represent an important constraint in the use of this type of SCP in aquafeed production [15, 16]. Furthermore, most of the nutrients present in yeast cells derive from within the cell. Therefore, several strategies have been developed to improve the digestibility of SCP products, such as by using mechanical disruption, autolysis, and enzymatic treatment [17]. Another limitation to the use of high levels of SCP in fish diets could be related to their high concentration in nucleic acids. The crude protein content of brewer’s yeast is about 46.5%, of which about 20% corresponds to nucleic acids (6–8% of total composition), mostly in the form of RNA [18]. However, unlike terrestrial animals in which an excess of dietary nucleic acids is toxic leading to an increase of plasma uric acid and metabolic disorders, fish seem to tolerate high levels of nucleic acids due to their efficient hepatic uricase activity [12, 19–21].

In addition to being a source of immune-stimulating compounds, dead yeasts have prebiotic properties, too. Indeed, dietary yeast extracts have been described to have positive effects in promoting the number of beneficial bacteria and inhibiting some pathogenic bacterial species in fish [22, 23]. For example, dietary supplementation of brewer’s yeast hydrolysate inhibited bacterial members of the genus *Mycoplasma*, and significantly increased *Cetobacterium* in the intestine of largemouth bass (*Micropterus salmoides*) [22]. However, except for a few studies, the impact of autolysed yeast on fish intestinal microbial communities is still unknown and scarcely investigated.

Accordingly, the present study aimed to investigate the effects of partial substitution of dietary fishmeal with 5% of either fish protein hydrolysate (FPH) or autolysed dried yeast (HiCell®, Biorigin, Brazil) on intestinal microbiota of gilthead sea bream (*Sparus aurata*). The FPH is produced from fish trim waste, and its nutritive value has been demonstrated in a number of marine fish species [24–26]. FPH is rich in free amino acids, bioactive compounds, and water-soluble proteins that improve feed palatability and digestibility. HiCell® is a commercial autolysed dried yeast obtained by the fermentation of a strain of *Saccharomyces cerevisiae* (GMO free). HiCell is suitable for animal feed use and it is assumed to have similar beneficial effects to FPH.

The high-throughput sequencing analysis of 16S rRNA gene fragments was used to assess the gut bacterial community composition of fish fed with different diets.

## Results

### Animal performance

Detailed information on fish growth performance and feed efficiency at the end of the feeding trial, have been recently reported by Fronte et al. [27]. Briefly, no statistically significant differences were observed in mortality, fish growth rate, and feed efficiency between dietary groups after 92 days of feeding trial.

### Gut microbiota composition

The microbiota of 45 samples collected from intestine were characterized by 16S rRNA gene amplicon sequencing on Illumina MiSeq platform. After data quality filtering, the run output was of 2,327,049 reads, which corresponded to an average number of 51,712 ± 15,620 (mean ± SD) reads per sample. A total number of 102 OTUs at 97% identity was identified in sea bream faecal samples collected at the end of the feeding trial. The Good’s coverage value was > 0.99 for all dietary groups, indicating that the number of identified OTUs accounted for the entire gut microbial communities (Table 1). All sequencing data were deposited as FASTQ files in the European Nucleotide Archive (EBI ENA) public database, under the accession code: **PRJEB35410**.

**Table 1:** Alpha diversity metrics of gut microbial communities in sea bream fed with three experimental diets

Items	Ctrl	DIETS	AY
		FPH	
Reads	52,248 ± 14,709	49,553 ± 17,950	53,334 ± 15,413
Observed OTUs	53.0 ± 4.2	47.4 ± 8.8	49.0 ± 6.1
Good’s coverage	0.99 ± 0.00	0.99 ± 0.00	0.99 ± 0.00
PD whole tree	6.1 ± 0.4^ab^	5.4 ± 1.0^b^	6.5 ± 0.9^a^
Chao1	56.2 ± 4.9	49.6 ± 9.4	52.5 ± 7.9
Shannon	1.8 ± 0.2^a^	1.7 ± 0.1^ab^	1.6 ± 0.2^b^
Simpson	0.5 ± 0.1	0.5 ± 0.0	0.5 ± 0.1

All data are reported as mean values (n = 15) ± SD. Different superscript letters on the same row indicate significant differences (*p* < 0.05).

**Fig. 1:**
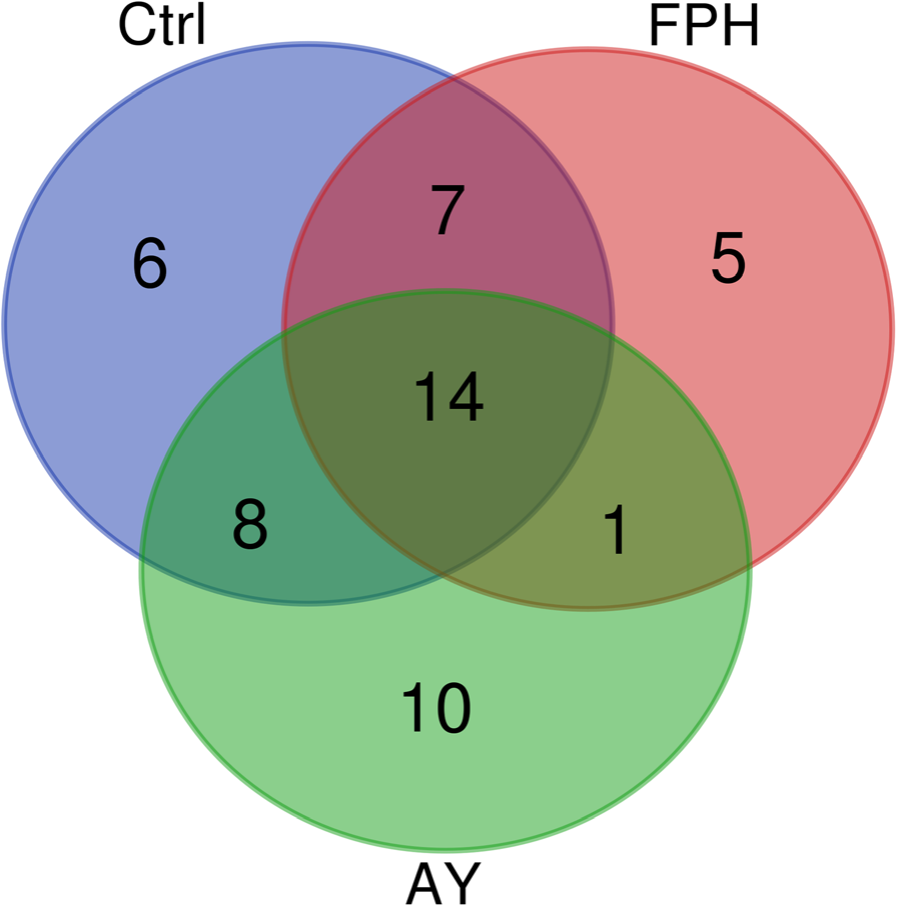
Venn diagram representing unique and shared Operational Taxonomic Units (OTUs) among all three dietary groups, regardless of diet

Fourteen OTUs constituted the core gut microbiota, i.e. those OTUs found in at least nine out of fifteen samples per group and shared regardless of the diet. Eight of them were assigned to *Firmicutes* represented by *Lactobacillus*, *Staphylococcus*, and *Bacillus* genera, and six to *Proteobacteria* phylum (Fig. 1).

**Fig. 2:**
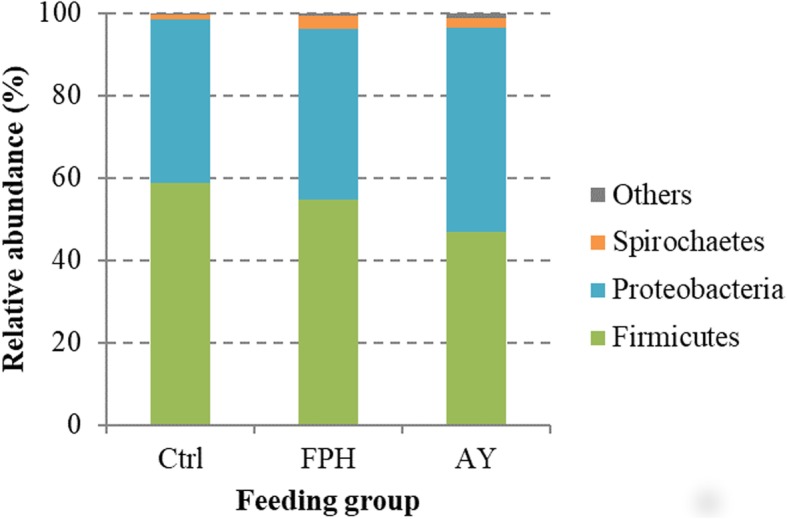
Relative abundance (%) of the overall most prevalent bacterial phyla in each dietary groups. In the figure, all taxa with an overall abundance of ≥1% were reported

**Fig. 3:**
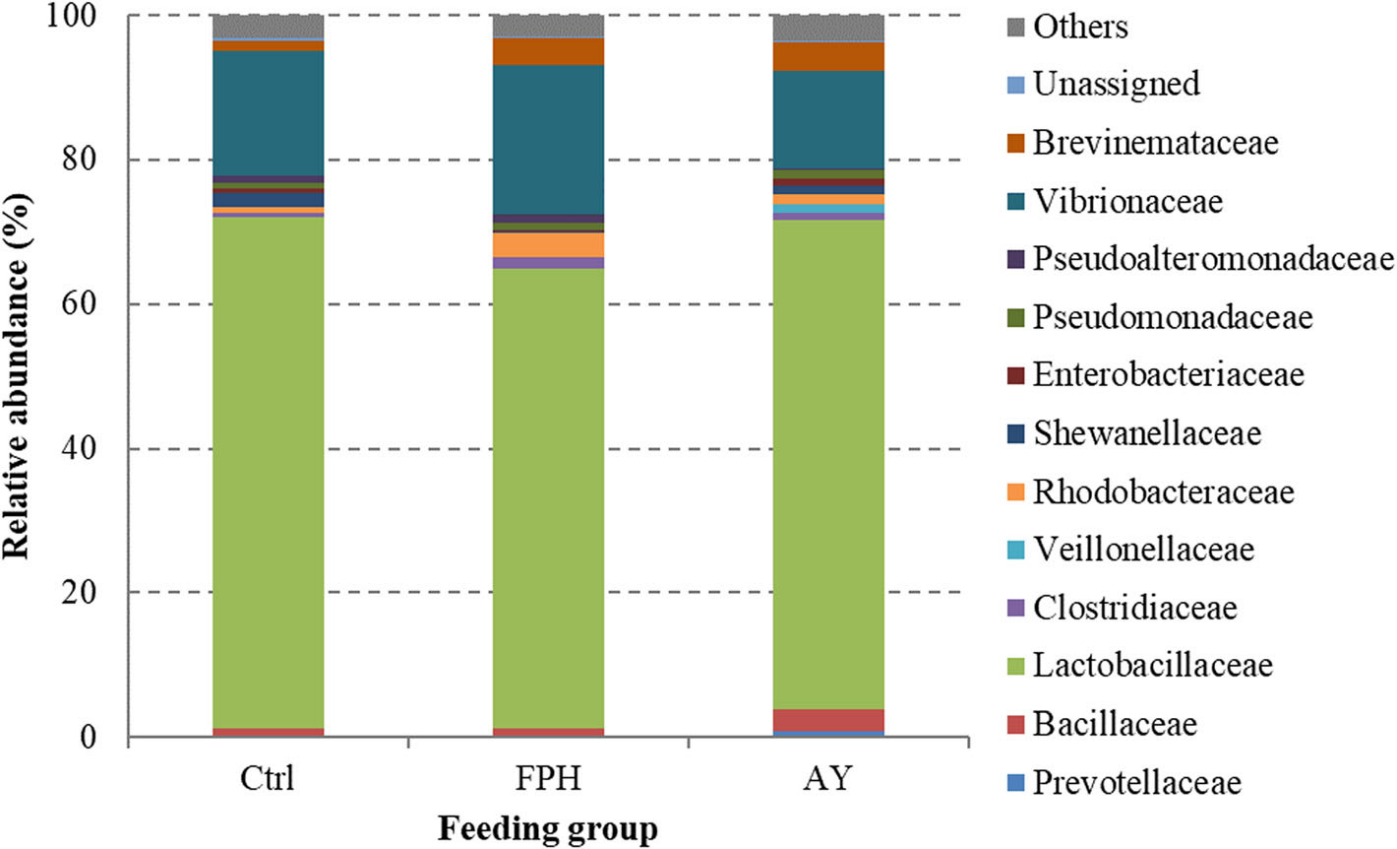
Relative abundance (%) of the overall most prevalent bacterial families in each dietary groups. In the figure, all taxa with an overall abundance of ≥1% were reported

**Fig. 4:**
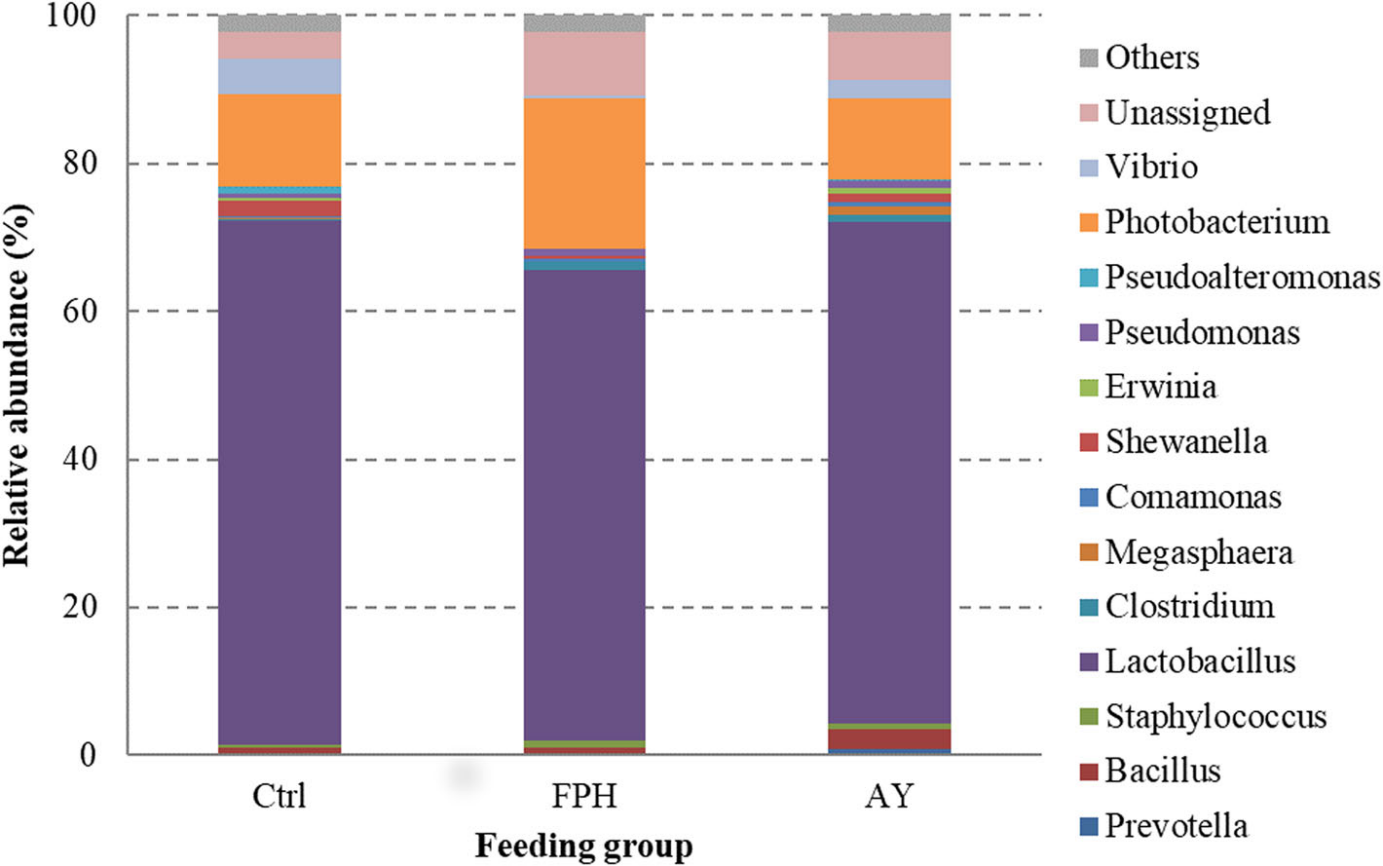
Relative abundance (%) of the overall most prevalent bacterial genera in each dietary groups. In the figure, all taxa with an overall abundance of ≥0.5% were reported

The microbial community profiles of each fish were outlined at the phylum, class, order, family, genus, and species. By taking into account all samples, the microbial community comprised 7 phyla, 10 classes, 19 orders, 29 families, 40 genera, and 19 species (Please see Additional file 1). *Cyanobacteria* phylum, essentially represented by *Streptophyta* order, was dominant in all samples counting more than 85% of reads. However, *Cyanobacteria* phylum, along with taxa assigned to chloroplast and mitochondria, were removed from the analysis being considered plant-derived sequences. After removing all eukaryotic OTUs and by considering only the most representative taxa, the overall intestinal bacterial community of sea bream consisted of 3 phyla, 5 classes, 9 orders, 12 families, 13 genera, and 4 species. The gut microbiome structure of each dietary group is shown at the phylum (Fig. 2), family (Fig. 3), and genus (Fig. 4) level. The taxa abundance at species level was unreliable because of the remarkable number of unassigned sequences that were found (78–85%); therefore it was excluded from the analysis.

To calculate alpha rarefaction indices, a sequencing depth of 19,500 reads per sample was taken into account; this value corresponded to the minimum number of reads found in our samples. Among alpha diversity metrics, both phylogenetic diversity “PD whole tree index” and “Shannon diversity index” resulted significantly lower in comparison to control group in sea bream fed with FPH diet, but not in AY feeding group (Table 1). On the contrary, dietary fishmeal replacement with fish protein hydrolysate or autolyzed yeast did not affect species richness, which resulted quite low regardless of the diet, as indicated by “Chao 1” and “Observed OTUs” values (Table 1).

**Fig. 5:**
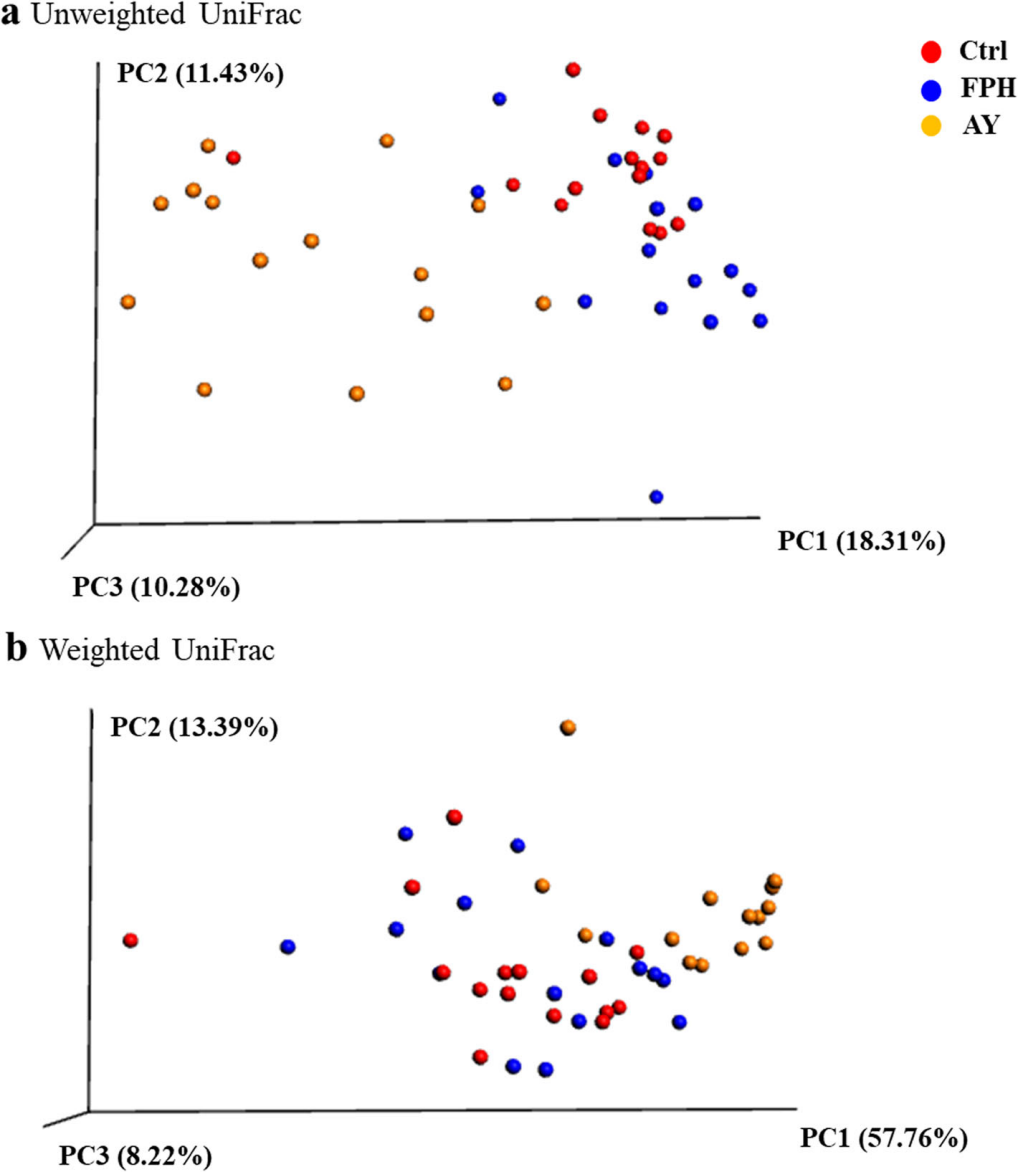
Beta diversity metrics. Principal coordinate analysis (PCoA) of unweighted (**a**) and weighted (**b**) Unifrac distances of gut microbial communities associated to different diet. The figures show the 3D plot of individual fish according to their microbial profile at genus level

Changes in beta-diversity, i.e. between microbial communities, were found both in type (unweighted UniFrac) and abundance (weighted UniFrac) of taxa. As displayed in unweighted (Fig. 5a) and weighted UniFrac PCoA (Fig. 5b) plots, PC1 and PC2 together explained 30 and 71% of the variation between individuals, respectively. In both plots, fish fed with AY diet clearly clustered separately from the control and FPH groups (Fig. 5a, b). Outcomes of multivariate analysis were strongly validated (*p* = 0.001) by nonparametric permutation test Adonis and ANOSIM. Results of pairwise statistical analysis were summarized in Table 2.

**Table 2:** Results of Analysis of similarity (ANOSIM) and permutational multivariate analysis of variance (Adonis) based on Unweighted and Weighted UniFrac distances

	Unweighted		Weighted	
**ANOSIM**				
	*p* value	R value	*p* value	R value
Ctrl vs FPH	**0.004**	0.144	0.199	0.028
Ctrl vs AY	**0.001**	0.514	**0.001**	0.489
FPH vs AY	**0.001**	0.480	**0.001**	0.341
**Adonis**				
	*p* value	R^2^	*p* value	R^2^
Ctrl vs FPH	**0.001**	0.08	0.285	0.04
Ctrl vs AY	**0.001**	0.19	**0.001**	0.38
FPH vs AY	**0.001**	0.18	**0.001**	0.30

Significant *p*-values (*p* < 0.05) are reported in bold.

### Dietary modulation of gut microbiota

The gut microbiota of fish in the present study was mainly dominated, regardless of the diet, by three phyla: *Firmicutes* (41–58%), *Proteobacteria* (40–49%), and to a lesser extent *Spirochaetes* (1–3%) (Fig. 2). As expected from diet formulations, which were practically equivalent in terms of vegetables and animal ingredients, *Firmicutes:Proteobactera* ratio was similar between the feeding groups. Although no differences were found at phylum level between feeding groups, Kruskal-Wallis analysis revealed significant changes in the relative abundance of bacteria at lower phylogenetic levels (Table 3). Major differences were found between fish fed with AY diet and control fish group. At order level, indeed, diet AY caused an enrichment in *Bacillales* (*p* < 0.01) and *Clostridiales* (*p* < 0.05) in comparison to the control group, whereas fish fed with diet FPH showed a significantly lower (*p* < 0.001) amount of bacteria belonging to *Alteromonadales* and *Enterobacteriales* than the other two feeding groups (Table 3).

**Table 3:** Percentages of the most abundant taxa (mean ± SEM) found in all dietary groups

Phylum	Ctrl			FPH			AY			***p***-value
*Firmicutes*	58.6	±	2.9	54.6	±	4.3	41.5	±	2.9	
*Proteobacteria*	39.7	±	3.1	41.5	±	4.5	49.4	±	2.9	
*Spirochaetes*	1.1	±	1.1	3.3	±	2.8	2.5	±	1.2	
**Class**										
*Bacilli*	73.5	±	3.9	66.2	±	6.2	72.2	±	3.9	
*Clostridia*	1.0	±	0.2	2.1	±	0.6	2.5	±	0.7	
*Alphaproteobacteria*	1.2	±	0.3^b^	3.6	±	1.8^b^	1.8	±	0.6^a^	*
*Gammaproteobacteria*	21.9	±	4.1	23.3	±	6.0	17.1	±	4.5	
*Brevinematae*	1.5	±	1.4	3.8	±	3.3	4.0	±	1.8	
**Order**										
*Bacillales*	1.9	±	0.2^b^	2.3	±	0.4^ab^	3.7	±	0.5^a^	**
*Lactobacillales*	71.5	±	3.9	63.9	±	6.1	68.4	±	3.6	
*Clostridiales*	< 1.0^b^			2.0	±	0.6^ab^	2.5	±	0.6^a^	*
*Rhodobacterales*	< 1.0			3.3	±	1.7	1.3	±	0.6	
*Alteromonadales*	2.0	±	0.5^a^	< 1.0^b^			1.1	±	0.2^a^	***
*nterobacteriales*	0.6	±	0.2^ab^	< 1.0^b^			1.1	±	0.2^a^	***
*Pseudomonadales*	0.8	±	0.2	1.0	±	0.2	1.0	±	0.2	
*Vibrionales*	18.2	±	4.1	21.9	±	6.1	13.6	±	4.6	
*Brevinematales*	1.4	±	1.4	3.8	±	3.2	3.9	±	1.2	
**Family**										
*Prevotellaceae*	< 0.5^b^			n.d.			0.8	±	0.3^a^	***
*Bacillaceae*	1.1	±	0.2^b^	1.2	±	0.4^b^	2.8	±	0.5^a^	**
*Lactobacillales*	70.9	±	3.9	63.6	±	6.1	67.8	±	3.7	
*Clostridiaceae*	0.5	±	0.0	1.6	±	0.6	1.0	±	0.5	
*Veillonellaceae*	n.d.			n.d.			1.2	±	0.2	***
*Rhodobacteriaceae*	0.7	±	0.3	3.30	±	1.8	1.3	±	0.6	
*Shewanellaceae*	2.0	±	0.5^a^	< 1.0^b^			1.1	±	0.2^a^	***
*Enterobacteriaceae*	0.6	±	0.2^ab^	< 1.0^b^			1.1	±	0.2^a^	***
*Pseudomonadaceae*	0.7	±	0.1	1.0	±	0.2	1.0	±	0.2	
*Pseudoalteromonadaceae*	1.0	±	0.2^a^	1.1	±	1.0^b^	< 0.5^b^			**
*Vibrionaceae*	17.2	±	4.1	20.8	±	5.9	13.5	±	4.6	
*Brevinemataceaeae*	1.4	±	1.4	3.8	±	3.2	4.0	±	1.8	
**Genus**										
*Prevotella*	n.d.			n.d.			0.8	±	0.3	***
*Bacillus*	0.9	±	0.2^b^	1.5	±	0.4^b^	2.3	±	0.6^a^	**
*Staphylococcus*	< 0.5			0.8	±	0.1	0.8	±	0.2	
*Lactobacillus*	70.9	±	3.9	63.6	±	6.1	67.8	±	3.7	
*Clostridium*	< 0.5			1.3	±	0.6	0.8	±	0.5	
*Megasphaera*	n.d.			n.d.			1.2	±	0.3^a^	***
*Comamonas*	< 0.5			< 0.5			0.6	±	0.2	
*Shewanella*	2.0	±	0.5^a^	< 0.5^b^			1.1	±	0.3^a^	***
*Erwinia*	< 0.5^ab^			< 0.5^b^			0.6	±	0.2^a^	**
*Pseudomonas*	0.7	±	0.1	0.9	±	0.3	1.0	±	0.3	
*Pseudoalteromonas*	0.9	±	0.2^a^	< 0.5^b^			< 0.5^b^			***
*Photobacterium*	12.4	±	4.2	20.2	±	5.8	10.9	±	4.4	
*Vibrio*	4.7	±	1.3^a^	0.5	±	0.2^b^	2.5	±	0.7^a^	***

“n.d.” means not detected. Statistical significance: (*) *p* < 0.05; (**) *p* < 0.01; (***) *p* < 0.001

Different superscript letters indicate statistically significant differences (Dunn’s post hoc test, *p* < 0.05)

Diet AY was associated with an increased proportion of *Prevotellaceae*, *Bacillaceae*, *Veillonellaceae* families (Table 3, Fig. 3). Bacteria belonging to *Shewanellaceae* and *Enterobacteriaceae* families were negatively affected by dietary inclusion of hydrolysed fish protein (*p* < 0.001). Contrariwise, irrespective of diet type, *Lactobacillaceae,* constituted the most abundant bacterial family (63–70%) found in our samples (Table 3, Fig. 3). Accordingly, *Lactobacillus* was the most numerous genus in all dietary groups, followed by *Photobacterium* (11–20%), mainly represented by *Photobacterium damselae* species (Fig. 4). However, the most noticeable difference was that of *Prevotella* and *Megasphera* genera. In particular, they were detected only in the gut microbiota of fish fed with AY diet (Fig. 4). In the same dietary fish group, higher abundance of bacteria assigned to genus *Bacillus* (2.3%) was found. Conversely, diet FHP led to a significant decrease of bacteria belonging to *Shewanella* genus (Table 2). Gut microbiota of fish fed with control diet, was characterized by higher percentage of *Pseudoalteromonas* genus than microbiota of FHP and AY dietary groups, wherein this bacterial genus resulted almost undetectable (< 0.5%) (Table 3). Lastly, dietary fish protein hydrolysate supplementation caused a significant decrease (*p* < 0.001) of bacteria assigned to *Vibrio* genus (Table 3). The between-group differences were also tested to compare the mean relative abundances of individual OTUs. Statistically significant changes were mainly found between AY group and the other two feeding groups. The results of Fisher’s test are reported in Additional file 2. Relative abundance of 11 OTUs was significantly influenced by the diet (*p* < 0.05). Interestingly, five of them were assigned to *Lactobacillus* genus.

## Discussion

The use of plant protein sources to replace fishmeal is a major trend in aquafeeds. However, the inclusion of plant-derived materials in the diets for carnivorous fish species is limited by their nutritional deficiencies and aminoacid imbalances, and by the presence of various antinutritional factors [28].

*S. cerevisiae* is the most common single cell protein source used as supplement in aquafeeds, due to its relatively high protein, energy, and micronutrient content. Furthermore, yeast is commonly non-pathogenic, free of plasmid-encoded antibiotic resistance genes and resistant to bile and acidic pH. There are several evidences demonstrating that dietary yeasts improve fish growth performance and feed efficiency, as well as enhance gut mucosal surface in fish [29–32]. Yeast could be also considered as a functional ingredient since it contains several immune-stimulating compounds, such as β-glucans and mannan-oligosaccharides, which positively influence immune responses and stress tolerance of fish [8, 9, 33]. In the present study, the replacement of FM with 5% of either FPH or yeast autolysate did not ameliorate or worsen the growth performances of sea bream. However, at the end of the feeding trial, intestine of fish fed with the HiCell® (Biorigin, Brazil) supplemented diet showed a higher goblet cell density and increased nutrient absorbing area [27]. Similarly, dietary yeast hydrolysate improved the antioxidant ability and enhanced the immune response of largemouth bass (*Micropterus salmoides*) without any negative impact on growth [33].

Our results clearly indicated that dietary yeast in the form of dried yeast autolysate, modulated fish intestinal microbiota by promoting the proliferation of some beneficial microorganisms. This is in line with previous studies reporting that low levels (1–2%) of dietary brewer’s yeast might affect fish intestinal microbial communities [34–36]. Indeed, when yeasts were used as live feed in rainbow trout (*Oncorhynchus mykiss*), there was a positive modulation of gut microbiota with an increased amount of lactic acid bacteria [29]. However, to our knowledge, the present study is the second investigation on this topic that used a high-throughput sequencing technique (i.e. Illumina MiSeq platform) and the first study on gilthead sea bream.

Results obtained from our metabarcoding analysis indicated that the most abundant phyla in sea bream intestine, regardless of the administered diet, were *Firmicutes* and *Proteobacteria*. In line with our previous studies, these phyla usually represent up to 90% of fish intestinal microbiota in both marine, and freshwater species [37–39]. Unlike the data already reported in literature about gut bacterial community of gilthead sea bream [37, 40–42], *Actinobacteria* and *Bacteriodetes* were scarcely represented in our samples. This could be partly due to the high percentage of *Streptophyta*, deriving from undigested feed, which negatively affected the detection of other less abundant taxa. For the same reason, we cannot exclude that the species richness could be underestimated in our samples, as the expected number of OTUs is usually higher in sea bream intestine [37, 40, 41]. However, the sequencing coverage for all the dietary groups was more than 99.9% indicating that the OTUs found in our samples were representative of the sampled population.

As expected from feed formulations with similar proportion of vegetable and animal ingredients, the *Firmicutes*:*Protobacteria* ratio was comparable between dietary groups. Indeed, *Firmicutes* and *Proteobacteria* phyla are usually discriminatory for diet type being the dominance of *Firmicutes* more related to diets with plant ingredients than to fishmeal-based diets [39, 43–45]. Although we did not observe an overall effect of HiCell® dietary supplementation on fish gut bacterial richness, the bacterial diversity in fish fed AY diet was reduced in comparison to the control group, whereas no decrease in Shannon’s diversity index value was observed in fish fed with 5% of fish protein hydrolysate. In line with our study, the diversity tended to decrease with the increase of dietary brewer’s yeast hydrolysate inclusion level in largemouth bass (*Micropterus salmoides*) [22]. Contrariwise, no significant differences in bacterial richness and diversity were found in gut microbiota of Arctic charr (*Salvelinus alpinus*) when 40% of dietary fishmeal was replaced with either intact or extracted yeast cells [46].

Usually a reduction in diversity is considered a negative effect as it leads to less competition for opportunistic or invading pathogens, which could thus easily colonize the gastrointestinal tract of fish [47].

Although we did not observe any effect of 5% fishmeal replacement with alternative nitrogen sources on intestinal bacterial phylum profile, at lower taxonomical levels the composition of gut microbiota, in terms of relative abundance of specific taxa, was significantly influenced by the dietary treatment. Multivariate analysis of the microbial communities showed that fish fed autolysate yeast diet clustered separately from the control group. In particular, dietary yeast led to an enrichment in bacteria belonging to *Prevotellaceae*, *Bacillaceae*, and *Veillonellaceae* families, whereas *Lactobacillaceae*, mainly represented by genus *Lactobacillus*, constituted the largest percentage (more than 60%) of the intestinal microbiota of all sea bream, irrespective to the diet. This result supports the thesis that lactobacilli are part of the natural gut microbiota of several finfish species [48]. Lactic acid bacteria are generally recognised as beneficial microorganisms associated with a healthy intestine and are often used as probiotics in livestock rearing and fish culture practices; therefore, an increase in their number is considered desirable [49–51]. Indeed, it was expected that components of yeast cell wall (beta-glucans, mannan-oligosaccharides, and chitin) would have acted as prebiotics by providing favourable conditions for growth of *Lactobacillus*. Increased *Lactobacillus* levels were also found in intestine of rainbow trout and Arctic char fed a diet supplemented with a probiotic mixture of lyophilized or extracted yeast [29, 46]. On the other hand and in line with our findings, Zhou and colleagues [22] found only little effects on the abundance of lactobacilli in intestine of largemouth bass fed a diet supplemented with brewer’s yeast hydrolysate.

Interestingly, only the intestine of sea bream fed with yeast harbored members of genera *Prevotella* and *Megasphera*. Bacteria of genus *Prevotella* are known for their ability to degrade complex plant polysaccharides; indeed in human, these bacteria have been clearly associated to plant-based diets, which are rich in fibers [52, 53].

Members of *Megasphaera* genus are producers of short chain fatty acids (SCFAs) [54]. Acetate, propionate, and butyrate are the most abundant SCFAs in the gastrointestinal tract of fish and other vertebrates. Among the microbial-derived SCFAs, butyrate is the most important due to its several well documented positive effects on the intestinal health [55–57]. In particular, butyrate can act through both local and systemic pathways, serving as energy substrate or signaling molecule, thus affecting satiety, energy production, and storage, and exerting a number of anti-inflammatory effects [58]. Therefore, dietary modulation of SCFAs production in fish intestine should be a desirable goal. HiCell® dietary inclusion seemed to promote also the growth of bacteria assigned to *Bacillus* genus. This genus is a member of *Firmicutes* phylum and includes both, pathogenic and beneficial bacterial species. Among probiotic candidates, *Bacillus subtilis* has been widely assayed in fish. Numerous studies have demonstrated that its administration enhances immune response and disease resistance [59–61].

Contrariwise, fish protein hydrolysate led to a significant decrease of bacteria belonging to *Shewanella* genus. *Shewanella* genus includes several species known to produce omega-3 fatty acids (eicosapentaenoic acid and docosahexaenoic acid) [62]. For this reason, they have been used as probiotics in fish culture practices [63, 64]. Therefore, the observed reduction of bacteria assigned to *Shewanella* genus in fish fed with FPH diet should be considered an adverse effect. Contrariwise, bacterial genus *Pseudoalteromonas*, which was well represented in gut microbiota of control fish fed with a fishmeal-based diet, could be successfully used as a probiotic in animal farming. As reported in previous studies, *Pseudoalteromonas* species have indeed the ability to reduce competing microbiota [65–67]. In shrimp (*Penaeus vannamei*), it was demonstrated that *Pseudoalteromonas* probiotic mechanism consists in the production of bioactive compounds with antibacterial, antifouling and antibiofilm activities [68].

In summary, this is the first metabarcoding characterization of the gut microbiome of sea bream fed with a basal diet with partial substitution of fishmeal with 5% of either fish protein hydrolysate (FPH) or commercial brewer’s yeast autolysate (HiCell®, Biorigin). This analysis revealed a clear intestinal microbiota modulation in response to autolyzed yeast dietary inclusion. In particular, the abundance of some beneficial bacteria, i.e. indigestible carbohydrate degrading and SCFA producing bacteria, was positively affected. Therefore, this study provides the first indication that brewer’s yeast autolysate could be a valid alternative to FM protein source as well as a valid functional ingredient for aquafeed production.

## Methods

### Feeding trial and sample collection

Details of the experimental design and feeding protocol have been recently described by Fronte et al. [27]. Briefly, the trial was set at the Experimental Center of VRM srl farm, located in Civitavecchia (Italy). A total number of 720 gilthead sea bream (*Sparus aurata*), purchased from the farm “Valle Ca’ Zuliani”, Italy (lot number SA27062015), were randomly distributed into nine circular fibreglass tanks of 2000 L. Fish were acclimatized for 1 week under natural photoperiod and fed to visual satiety with a standard commercial diet (Naturalleva, VRM srl, Italy). After the acclimation period, fish were fed ad libitum twice per day for 92 days with three different diets in triplicate (3 tanks/diet). Three experimental feeds were formulated by Naturalleva VRM srl (Cologna Veneta VR, Italy). In particular, a feed based on FM/vegetable meal (containing 46% crude protein and 16% fat) was used as the control diet (Ctrl). The other two feeds were formulated by replacing FM with 5% of either fish protein hydrolysate (FPH) or autolyzed yeast (AY) (HiCell®) that was produced by Biorigin (Brazil) and supplied by Albitalia Srl (Milan, Italy). The proximate composition and the principal components of all tested diets are reported in Table 4.

**Table 4:** Diet formulation and proximate composition (modified from Fronte et al. [27])

	DIETS		
	Ctrl	FPH	AY
**Ingredients (% as it is):**			
Fishmeal	22.25	17.80	17.80
Corn gluten meal	17.80	16.91	17.71
Guar germ meal	15.13	14.03	17.68
Soybean meal	10.70	10.68	10.68
Soy Protein Concentrate	9.38	9.79	10.04
Wheat middling	7.45	8.90	7.12
Fish oil 92	6.93	6.93	6.93
Fish protein hydrolysed		4.60	
HiCell® – autolysed yeast			4.60
Pea meal	4.45	4.45	1.53
Cameline oil	2.42	2.42	2.42
Mineral/Vitamin supplement	2.00	2.00	2.00
Rapeseed oil	1.49	1.49	1.49
**Proximate composition (% as it is):**			
Crude protein	46.00	46.10	46.10
Crude fat	16.20	16.10	16.10
Crude fibre	2.10	2.00	2.20
Ash	6.20	5.80	6.00
Gross Energy (MJ/kg)	18.70	18.80	18.60

At the end of feeding trial 15 fish/dietary group (5 fish/tank) were sacrificed and used for sample collection. For this, fish were caught and immediately euthanized with an overdose of natural clove oil containing the ingredient eugenol (Guinama S.L; Spain, Ref Mg83168). Clove oil was added at a dose of 400 mg/l to water, and premixed so that all of the oil was emulsified. Fish were then transferred to this water, loosing consciousness within seconds and ceasing breathing quickly. Since the time in which fish succumb to hypoxia and die, can vary in timescale from fish to fish, fish were left in the solution for 15 min to confirm their death. The lack of operculum movement for 15 min provided confirmation of death before disposal of the animal. Dead fish were removed with the aid of a net from the anaesthetic solution and placed on a sterile white towel on their side. Then, intestine was aseptically removed from each fish and the faecal matter was obtained by squeezing out and scrapping the intestinal mucosa with a sterile spatula, in order to collect both, the digesta- and the mucosa-associated microbiota (transit- and resident microbiota). The mixed faecal and gut mucosa samples were quickly transferred into a sterile Eppendorf tube containing 800 μl of Xpedition™ Lysis/Stabilization Solution (Zymo Research, Irvine, CA, USA) and then stored at room temperature, until analysis.

### Microbial DNA extraction

The bacterial DNA was obtained from 200 mg of intestinal matter by automated extraction using Cador Pathogen 96 QIAcube HT Kit and the QIAcube HT instrument (Qiagen, Italy), following the manufacturer’s instructions with few modifications. The Pathogen Lysis Tubes containing samples were pre-treated by means of a TissueLyser II (Qiagen) for 2 min at 25 Hz. The extracted DNA was quantified using a NanoDropTM 2000 Spectrophotometer (Thermo Scientific, Milan, Italy) and then stored at − 20 °C until analysis.

### Preparation of 16S amplicon library and sequencing

The complete protocol for 16S rRNA gene library preparation and sequencing has been described in Rimoldi et al. [69]. Briefly, the 16S amplicon libraries were prepared using the tailed forward and reverse primer Pro341F (5′-CCTACGGGNBGCASCAG-3′) and Pro805R (5′-GACTACNVGGGTATCTAATCC-3′), specific for V3–V4 region of bacterial 16S rRNA gene [70]. The expected size of PCR amplicons on Agilent 2100 Bioanalyzer trace was ~ 550 bp. For libraries generation, the Illumina protocol “16S Metagenomic Sequencing Library Preparation for Illumina MiSeq System” (#15044223 rev. B) was applied. Nextera XT Index Kit (Illumina, San Diego, CA, USA) was used to incorporate two unique indexes to the 16S amplicons. All indexed paired-end libraries were quantified by qPCR-based quantification using KAPA Library Quantification Kits Illumina® Platforms (Kapa Biosystems Ltd., London, UK); then they were equimolar pooled for multiplexed sequencing and diluted to six picomolar. Thepooled libraries were then sequenced on an Illumina MiSeq platform (Illumina, San Diego, CA, USA) using v3 chemistry and 2 × 300 bp reads.

### Metabarcoding raw data analysis

Metabarcoding sequencing raw data (FASTQ format) were processed using the open-source bioinformatics pipeline QIIME v1.9.1 [71], at the default setting. Detailed description of data handling has been reported in Terova et al. [38]. Briefly, FLASH v1.2.11 software (http://sourceforge.net/projects/flashpage) was used to merge the overlapping paired-end reads. In the filtering process, all the sequences whose quality score (Q) was < 30 were discarded. A sequence similarity threshold of 97% was set to assign reads to OTUs and only the OTUs that represented at least 0.005% of total reads were retained. Greengenes database v.13.8 (http://greengenes.lbl.gov) was used as reference for taxonomy assignment. The resulting OTU tables were built using the custom script ‘summarize_taxa_through_plots.py’. All sequences assigned to the phylum *Cyanobacteria* (class *Chloroplast*), *Rickettsiales* order, and to *Mitochondria* family were removed from the analysis as they were considered plant contaminants.

Alpha and beta diversity statistics have been performed using QIIME scripts ‘alpha_rarefection.py’ and ‘jackknifed_beta_diversity_.py’, respectively [39]. Good’s coverage, observed OTUs, Chao1 index, PD whole tree, Shannon and Simpson diversity indices were calculated. Both weighted (presence/absence/abundance matrix) and unweighted (presence/absence matrix) UniFrac beta diversity distance matrices were calculated [72, 73] and visualized by Principal Coordinates Analysis (PCoA). A Venn diagram displaying the core microbiome (OTUs shared regardless of the diet and found in at least nine out of the fifteen samples per dietary group) was drawn using the web tool http://bioinformatics.psb.ugent.be/webtools/ Venn/.

### Statistics

Normality and homogeneity of variance of data were checked by Shapiro-Wilk and Levene’s test, respectively. To test null hypothesis (p < 0.05), one-way ANOVA followed by Tukey-Kramer post hoc test or nonparametric Kruskal-Wallis and Dunn’s post hoc test were performed depending on normality and homoscedasticity of the data. To perform statistics on microbial relative abundance data, the percentage values were firstly square root -transformed. Only those taxa with an overall abundance of more than 1% (up to family level) and 0.5% at genus level were considered for the analysis. Differential abundance analysis of OTUs between groups was performed using MetagenomeSeq (R package) applying Fisher’s test with False Discovery Rate (FDR) correction (p < 0.05).

The significance of the calculated beta-diversity dissimilarities was assesed by non-parametric analysis of similarities (ANOSIM) and Adonis tests based on 999 permutations using QIIME script ‘compare_categories.py’.

## Supplementary information

**Additional file 1:.** List of OTUs and corresponding number of reads found in sea bream intestine

**Additional file 2:.** List of OTUs significantly influenced by diet.

## Data Availability

The dataset generated corresponding to the final 16S rRNA gene sequences is available in the European Nucleotide Archive (ENA), accession code PRJEB35410.

## Abbreviations

ANOSIM: Analysis of similarities
AY: Yeast autolysate
Ctrl: Control
FM: Fishmeal
FPH: Fish protein hydrolysed
OTU: Operational taxonomic unit
PCoA: Principal coordinate analysis
SCFA: Short chain fatty acid
SCP: Single cell protein
