# Correction to: Effects of hydrolyzed fish protein and autolyzed yeast as substitutes of fishmeal in the gilthead sea bream (*Sparus aurata*) diet, on fish intestinal microbiome

**DOI:** 10.1186/s12917-020-02416-1

**Published:** 2020-06-29

**Authors:** S. Rimoldi, E. Gini, J. F. A. Koch, F. Iannini, F. Brambilla, G. Terova

**Affiliations:** 1 Department of Biotechnology and Life Sciences, University of Insubria, Via J.H. Dunant, 3, 21100 Varese, Italy; 2 Biorigin Brazil, Rua XV de Novembro, 865, Lençóis Paulista, São Paulo, 18680-900 Brazil; 3 VRM srl Naturalleva, Via Sommacampagna, 63/D, 37137 Verona, Italy

**Correction to: BMC Vet Res 16, 118 (2020)**

**https://doi.org/10.1186/s12917-020-02335-1**

The original article [1] contains errors in the following two passages of text:

1. The following text in the Conclusion sub-section of the **Abstract**:
  ‘Brewer’s yeast autolysate could be a valid alternative protein source to FM as well as a valid functional ingredient for aquafeed production.’
  This should instead state the following:
  ‘Autolysed dried yeast obtained by the fermentation of a strain of *Saccharomyces cerevisiae* could be a valid alternative protein source to FM as well as a valid functional ingredient for aquafeed production.’
2. The following text in final paragraph of the **Discussion** section:
  ‘In summary, this is the first metabarcoding characterization of the gut microbiome of sea bream fed with a basal diet with partial substitution of fishmeal with 5% of either fish protein hydrolysate (FPH) or commercial brewer’s yeast autolysate.’
  This should instead state the following:
  ‘In summary, this is the first metabarcoding characterization of the gut microbiome of sea bream fed with a basal diet with partial substitution of fishmeal with 5% of either fish protein hydrolysate (FPH) or commercial Autolysed dried yeast *Saccharomyces cerevisiae* (HiCell®, Biorigin).’
